# Hypoxic resistance of KRAS mutant tumor cells to 3-Bromopyruvate is counteracted by Prima-1 and reversed by N-acetylcysteine

**DOI:** 10.1186/s12885-016-2930-9

**Published:** 2016-11-18

**Authors:** Andrea Orue, Valery Chavez, Mary Strasberg-Rieber, Manuel Rieber

**Affiliations:** IVIC, Tumor Cell Biology Laboratory, Apartado 21827, Caracas, 1020A Venezuela

**Keywords:** Hypoxia, ALDH1A1, GLUT1, p53 reactivation, KRAS mutation

## Abstract

**Background:**

The metabolic inhibitor 3-bromopyruvate (3-BrPA) is a promising anti-cancer alkylating agent, shown to inhibit growth of some colorectal carcinoma with KRAS mutation. Recently, we demonstrated increased resistance to 3-BrPA in wt p53 tumor cells compared to those with p53 silencing or mutation. Since hypoxic microenvironments select for tumor cells with diminished therapeutic response, we investigated whether hypoxia unequally increases resistance to 3-BrPA in wt p53 MelJuso melanoma harbouring (Q61L)-mutant NRAS and wt BRAF, C8161 melanoma with (G12D)-mutant KRAS (G464E)-mutant BRAF, and A549 lung carcinoma with a KRAS (G12S)-mutation. Since hypoxia increases the toxicity of the p53 activator, Prima-1 against breast cancer cells irrespective of their p53 status, we also investigated whether Prima-1 reversed hypoxic resistance to 3-BrPA.

**Results:**

In contrast to the high susceptibility of hypoxic mutant NRAS MelJuso cells to 3-BrPA or Prima-1, KRAS mutant C8161 and A549 cells revealed hypoxic resistance to 3-BrPA counteracted by Prima-1. In A549 cells, Prima-1 increased p21CDKN1mRNA, and reciprocally inhibited mRNA expression of the SLC2A1-GLUT1 glucose transporter-1 and ALDH1A1, gene linked to detoxification and stem cell properties. 3-BrPA lowered CAIX and VEGF mRNA expression. Death from joint Prima-1 and 3-BrPA treatment in KRAS mutant A549 and C8161 cells seemed mediated by potentiating oxidative stress, since it was antagonized by the anti-oxidant and glutathione precursor N-acetylcysteine.

**Conclusions:**

This report is the first to show that Prima-1 kills hypoxic wt p53 KRAS-mutant cells resistant to 3-BrPA, partly by decreasing GLUT-1 expression and exacerbating pro-oxidant stress.

## Background

Tumor progression includes clonal selection of cells with mutated RAS or an inactive p53 tumor suppressor gene, leading to increased survival within the hypoxic tumor microenvironment. Aberrant signaling pathways induced by oncogenic KRAS mutations may help inactivate the functionality of the p53 tumor suppressor gene through critical effectors of oncogenic KRAS like Snail [1], Notch1 [2] or Ral GTPases [3]. Down-regulation of KRAS, RalB, and RalA increases p53 protein levels and results in a p53-dependent up-regulation of the expression of p21CDKN1A [3]. Prima-1 (2,2-bis(hydroxymethyl)-1-azabicyclooctan-3-one) like Prima-1 ^Met^/APR-246, belongs to a group of non-genotoxic small molecules that promote mutant p53 reactivation and significant growth inhibition in several human tumor cells [4–9]. More recently, these drugs were reported to activate wild-type p53 and induce apoptosis in wt p53 malignant melanoma tumors [7], and in hypoxic wt p53 breast cancer cells [8]. Prima-1^Met^ also has been shown to induce apoptosis in multiple myeloma [9], Ewing sarcoma irrespective of p53 status [10], in human prostate cancer, in a mouse leukemia cell line lacking p53 expression [11] and even in tumor cells lacking p53 through inhibition of thioredoxin reductase I [12]. A common mechanism to explain the loose dependence on p53 in the response to Prima-1 or Prima-1^Met^ could be that they take advantage of the high levels of oxidative stress common to tumor cells harbouring mutant p53 [8, 13] or oncogenic KRAS [14]. Supporting a role of oxidative stress in p53 reactivation, normoxic wt p53 breast cancer cells [8] and multiple myelomas [9] increase their susceptibility to Prima-1 with agents that impair the GSH/ROS balance like the glutathione antagonist, buthionine sulfoximine, which antagonizes cellular anti-oxidant defence [8, 9]. Reactive oxygen species (ROS) are also a byproduct of metabolism, being produced during electron transfer by high metabolic consumption in tumor cells with moderate ROS levels driving metabolic processes but high ROS promoting cell death [13, 14]. Oncogenic KRAS mutations increase ROS levels [14] and overexpression of GLUT1 in lung carcinomas [15]. This glucose receptor 1 (SLC2A1-GLUT1) transports glucose which has a role in antioxidant defense [16], since it is the first substrate in the pentose phosphate pathway generating NADPH, capable of donating electrons to antioxidant pathways to attenuate excessive oxidative stress [14–16]. Agents having anti-oxidant properties like pyruvate or N-acetylcysteine also counteract death by glucose depletion in human tumor cells [17]. Detoxification of stress caused by reactive lipid peroxidation can be helped by ALDH1A1 (EC 1.2.1.36) a putative cancer stem cell marker [18] belonging to a superfamily of NAD(P)^+^-dependent enzymes that catalyze the oxidation of a wide variety of endogenous and exogenous aldehydes to their corresponding carboxylic acids [18, 19]. ALDH1A1 has prognostic significance in non-small cell lung cancer [19]. In tumor progression, cancer cells adapt to hypoxic stress by inducing expression of genes coding for carbonic anhydrase IX (CAIX) [20–22] or vascular endothelial growth factor (VEGF) [23], which also are important targets in cancer therapy. As a redox-active transcription factor, the p53 protein core DNA-binding domain when in contact with DNA, can sense oxidative stress. When cells are exposed to Prima-1 or to Prima-1(MET), these molecules yield several active products among them methylene quinuclidinone (MQ), that reacts covalently to alkylate p53 cysteine residues and reactivate p53 function [6]. Moreover, MQ can also target cells irrespective of p53 by inhibiting thioredoxin reductase I and converting it to a pro-oxidant NADPH oxidase to further increase oxidative stress [6, 12]. Another potent pro-oxidant is 3-bromopyruvate (3-BrPA), a metabolic competitor of pyruvate [17], and an alkylating agent capable of depleting ATP and increasing metabolic stress by generating free radicals [24, 25]. 3-BrPA preferentially suppressed the growth of some colorectal carcinoma cells with KRAS or BRAF mutations which survived glucose starvation [26]. Since hypoxia [8] and some RAS mutations [26] may increase drug resistance partly by favouring p53 tumor suppressor dysfunction [8], this report investigated whether hypoxia unequally induces resistance to 3-BrPA in wt p53 tumor cells like MelJuso melanoma harbouring (Q61L)-mutant NRAS and wt BRAF, C8161 melanoma with (G12D)-mutant KRAS (G464E)-mutant BRAF and A549 lung carcinoma with a KRAS (G12S)-mutation. We also investigated whether the p53 reactivator, Prima-1 counteracts a possible hypoxic resistance to 3-BrPA. The rationale for studying Prima-1, which alkylates critical p53 thiol groups [6, 27] together with 3-BrPA, which alkylates key thiol groups in glycolytic and mitochondrial targets [24, 25], is because of their possible synergism to increase ROS [25, 26] and prevent proliferation and expression of genes associated with hypoxia and/or glycolysis in cells harbouring mutant RAS and a wt p53 gene.

## Methods

### Cell Lines

#### Human melanoma cells

- MelJuso cells are wt BRAF and mutated in NRAS- Q61L [28].
- C8161 cells were initially reported to be wild-type for both N-RAS and BRAF (http://www.wistar.org/lab/meenhard-herlyn-dvm-dsc/page/mapk-and-pi3k-pathways) with greater resistance to MEK inhibition in three-dimensional culture [29]. Quite recently, these cells were identified as having a G464E mutation in the BRAF P loop region, accompanied by an enhancing KRAS G12D mutation [30].

#### Non-small cell lung cancer cells

c) The A549 human lung adenocarcinoma cell line (www.atcc.org/~/ps/CRM-CCL-185.ashx) is being used as an *in vitro* model for non-small cell lung cancer (NSCL) harbouring a wt p53 gene and a KRAS gene mutation (p.G12S c.34G > A). These wt p53 NSCL cells were found to be resistant to a 24 h treatment with 100 μM Prima-1 under normoxia [31].

### Cell culture conditions and treatments under high glucose or physiological glucose

Sparse cells were allowed to attach to tissue-culture dishes for 20 h in high serum- glucose medium consisting of Dulbecco’s Modified Medium (DME) Sigma Cat # D1152 containing 4.5 g/lL glucose (∼23 mM) supplemented with 4 mM glutamine and 10% fetal calf serum. Treatments were added in this higher glucose medium for the indicated times. For studies in the low glucose medium, adherent cells seeded for 20 h in high serum- glucose medium were washed 3 times in isotonic phosphate-buffered saline pH 7.3, followed by addition of Dulbecco’s Modified Eagle’s Medium Sigma Cat # D5030, 5 mM physiological glucose, 2 mM glutamine and 5% dialyzed calf serum, together with other conditions indicated in each experiment [17]. Water-soluble reagents like Prima-1(Sigma #P0069) and/or 3-BrPA (Sigma Aldrich #238341) were freshly prepared [25], and added whenever indicated. Unequal time duration of experiments were chosen to harvest and analyze cells at different times, depending on whether earlier changes in RNA and protein, cell cycle events or overt cytotoxicity were studied.

### Hypoxia experiments

These were carried out in a hypoxic C-474 chamber equipped with Pro-Ox 110 oxygen controlling regulators (Biospherix, New York, N.Y.) to provide (≤2% oxygen).

### Relative cell viability/metabolic activity

This was estimated with Alamar Blue (resazurin) by measuring intracellular redox mitochondrial activity by quantitating the cell-catalyzed conversion of non-fluorescent resazurin to fluorescent resorufin [8]. Alamar Blue was added to a 10% final concentration to each one of 96 well plates after the appropriate treatment. This assay is valuable as an endpoint of proliferation or relative viability/metabolic activity. For these experiments, cells (5,000) were allowed to adhere overnight in 96 well TC plates. After the corresponding treatments, Alamar Blue (BioSource, Camarillo, CA, USA) was added without removing medium containing dead cells, and fluorescence measured 4 h later in a Fluoroskan Ascent microplate reader with an excitation of 544 nm and an emission of 590 nm. Standard deviations (S.D.) were used to determine a statistically significant difference in the octuplicate median values shown for metabolic activity/cell viability. Generally, S.D. results usually were within ±5% with a 95% statistical significance (*n* = 4). The criterion for statistical significance was taken as *p* < 0.05 by student *t* test, whenever indicated by *.

### High content cell cycle analysis by fluorescent imaging

This was carried out using the Cell Cycle Bio-Application algorithm provided with the Cellomics Arrayscan VTI at a magnification of 10X, used to identify objects by nuclear staining with Hoechst dye. A minimum of 500 individual cellular images or 20 fields were captured for each condition. The algorithm measured total nuclear intensity and selected for below 2n (subG1 dead cells), 2n (G1 cells), 2n-4n (S phase cells), 4 n (G2 cells) and above 4n DNA (multiplody or hypertetraploid cells) [32]. Generally, S.D. results usually were within ±5%.

### Intracellular ROS Quantitation

ROS intracellular generation was assayed in adherent A549 cells seeded in 96 well plates after 9 h of exposure to the indicated treatments in medium supplemented with 5 mM glucose. This was quantitated adding DCFH-DA (Life Technologies), a cell permeable non-fluorescent compound that can be hydrolyzed by intracellular esterases to DCFH, which fluoresces *green* when oxidized by H_2_O_2_. Cells were exposed for 30 min to 20 μM DCFH-DA and 20 μM LavaCell (Active Motif. Carlsbad, California 92008, USA) a *cell*-permeable, non-toxic compound that stains membranes of live cells *orange-red* emission (560-580 nM) for 30 min. Cell-associated fluorescence was determined in octuplicates, using the signal thresholding algorithms to identify fluorescence above the solution background from which fluorescent cells are identified in an Isocyte argon laser spectrofluorometer (Blueshift Biotechnologies, Inc., Sunnyvale, Ca.) identifying **ROS** in channel 1 green fluorescence (510–540) normalized to channel 3 orange-red cell fluorescence (560–580 nm).

### Crystal violet staining of surviving cells

Cells were subjected to the treatments indicated in each experiment. Surviving cells were evidenced following fixation in 90% ethanol and cell staining with 0.5% crystal violet (Cat # C-3886, Sigma–Aldrich, St. Louis, MO. 63103, USA) in 30% ethanol.

### Real-time and end-point RT-PCR

Cells were seeded in 5 cm-well plates (3 × 10^5^ cells per plate) in complete Dulbecco’s medium containing 20 mM glucose supplemented with 10% serum for 24 h. Cells were washed 3X with PBS and treated as indicated in medium supplemented with physiological 5 mM glucose and 5% dialyzed serum for 24 h. RNA extraction was performed using TRIZOL® (Life Technologies, Cat # 15596–026) and quantification was determined using a Qubit® 2.0 Fluorometer (Life Technologies, Cat #Q32866) with a Qubit™ RNA Assay Kit (Life Technologies, Cat # Q32852). The cDNA was prepared with the ProtoScript® First Strand cDNA Synthesis Kit (New England BioLabs, Cat # E6300S) using oligo dT as a primer. A GeneAmp® PCR System 9700 ABI machine was used for end-point PCR, followed by agarose gel electrophoresis, to confirm lack of reaction in the absence of template, and expected size of PCR products. All amplification reactions were prepared with Q5® High-Fidelity PCR Kit (New England BioLabs, Cat # E0555S). Real Time qPCR was carried out in an Illumina Eco Real-Time PCR machine, in reactions (10 μL) containing 5 μL KAPA SYBR FAST qPCR Master Mix (Kapa Biosystems), 0.5 μM of each primer pair, 1 μL of cDNA template (ng) and 1 μL RNAse-DNAse free water. PCR reactions were subjected to 95 °C for 3 min; followed by 40 cycles at 95 °C for 10 s and 60 °C for 30 s. This was followed by melting curve analysis. The primers sequences used described in Table 1, were obtained from Integrated DNA Technologies (IDT, Coralville, IA 52241, USA). In all cases, the expression of each gene was normalized by measuring the expression of the similarly treated housekeeping gene coding for actin (ACTN) or for glyceraldehyde-3-phosphate (GAPDH). All experiments were performed in triplicate. SigmaPlot 11.0 software was used for the statistics analysis of one-way analysis of variance or one-way ANOVA (*p* ≤0.01 or *p* ≤0.05 significance).

**Table 1:** Primer sequence for SYBR Green RT-qPCR and end point PCR analysis

Gen Name	Primer sequence Fw	Primer sequence Rv
ACTN	5´- CATGTACGTTGCTATCCAGGC-3´	5´- CTCCTTAATGTCACGCACGAT-3´
GAPDH	5´- GCACCACCAACTGCTTAGCA-3´	5´-TGGCAGTGATGGCATGGA-3´
SLC2A1 -GLUT1	5´- CGGGCCAAGAGTGTGCTAAA-3´	5´- TGACGATACCGGAGCCAATG-3´.
CAIX	5´- ATCCTAGCCCTGGTTTTTGG-3´	5´- GCTCACACCCCCTTTGGTT-3´
ALDH1A1	5´- CAAGATCCAGGGCCGTACAA-3´	5´- CAGTGCAGGCCCTATCTTCC-3´
LDHA	5´- ATCTTGACCTACGTGGCTTGGA-3´	5´- CCATACAGGCACACTGGAATCTC-3´
p21 CDK1N1	5´- GGACCTGGAGACTCTCA-3´	5´- CCTCCTGGAGAAGATCAG-3´

### Immunofluorescence staining

Immunofluorescence (IF) staining of cells was performed as previously described [33]. In brief, cells cultured on 96-well plates as indicated for each experiment, were washed with ice-cold PBS and fixed with 4% p-formaldehyde in phosphate-buffered saline. Cells were permeabilized with PBS containing 0.3% Triton X-100 and blocked in the same buffer adding 10 mg/ml bovine serum albumin and 1:1 dilution of mouse pre-immune serum. Subsequently, cells were incubated overnight with anti-human FITC-conjugated to GLUT1 monoclonal antibody MAB1418 clone 202915 diluted 1:8. and MAB 293 human VEGF mouse monoclonal antibody clone 26503, both from R&D (614 McKinley Place N.E. Minneapolis, MN55413 USA) followed by a 90 min incubation with Alexa Fluor 488-conjugated anti-mouse secondary antibody (Invitrogen). Examination of green GLUT1 was carried out in separate assays by fluorescence microscopy in which DNA containing nuclei were stained violet with Hoechst 33342. Cells showed no fluorescence after reaction with a negative control IgG in contrast to the reactivity seen with the specific monoclonal antibodies used.

## Results

### Prima-1 lowers SLC2A1-GLUT1 mRNA and protein expression and cooperates with 3-BrPA to promote toxicity against normoxic A549 cells

**Fig. 1:**
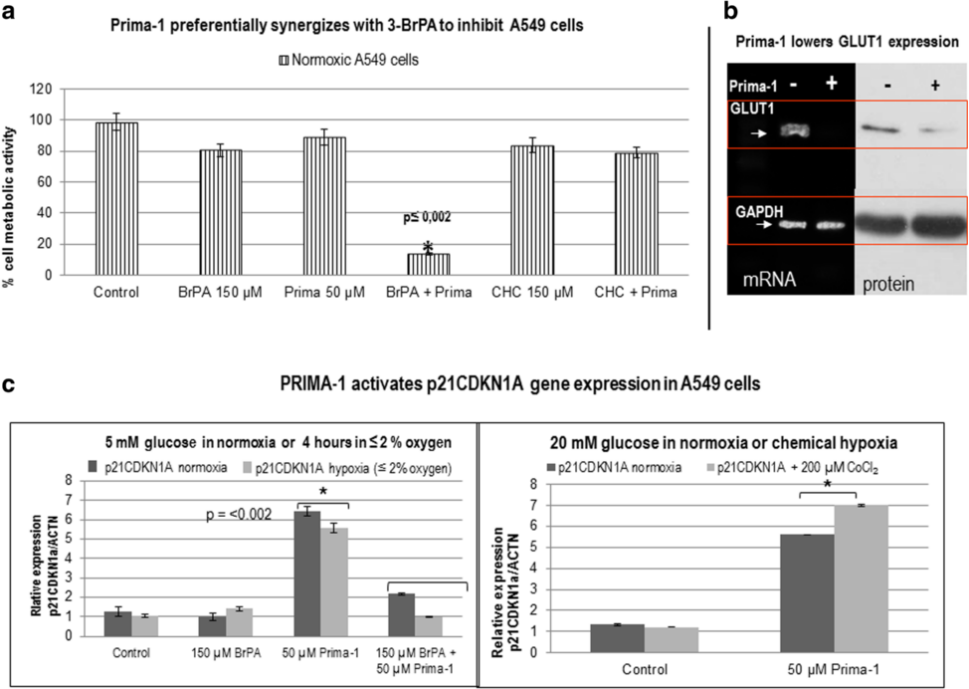
**a** 3-BrPA potentiates Prima-1 toxicity against A549 cells in 5 mM glucose. A549 cells (4X10^3^) were seeded in tissue culture 96 well plates in complete medium containing 20 mM glucose and 10% fetal bovine serum, then washed 3X with PBS and treated as indicated in each case, in medium supplemented with physiological 5 mM glucose, 2 mM glutamine and 5% dialyzed serum for 48 h. Relative proliferation /toxicity was assayed fluorometrically in octuplicate by the Alamar Blue method by quantitating the conversion of resazurin to fluorescent resorufin [8]. This revealed that 50 μM Prima-1 cooperated with 3-BrPA rather than with CHC to suppress A549 cell growth. **b** Prima-1 decreases SLC2A1-GLUT1 in A549 cells. Sparse cells were seeded in 5 cm tissue culture plates (5 **×** 10^5^cells per plate) in complete Dulbecco’s medium containing 20 mM glucose supplemented with 10% serum for 18 h, then washed 3X with PBS and treated as indicated in each case, in medium supplemented with physiological 5 mM glucose, 2 mM glutamine and 5% dialyzed serum whenever indicated (+) for 24 h. After RNA extraction with TRIZOL and quantification in a Qubit® 2.0 Fluorometer, cDNAs were prepared for end-point PCR analysis as indicated under Methods.essentially similar results were obtained in cells treated with Prima-1 in 5 mM glucose (not shown). Cells treated in parallel with those used for RNA analysis were used for GLUT1 protein immune blot [40]. **c** Prima-1 activates p21CDKN1A gene expression in A549 cells in 5 mM glucose. qPCR was used to determine relative expression of the p21CDK1N1 gene in control and treated cells, after RNA extraction, cDNA preparation and qPCR, as indicated under Methods. *denotes significance between treated cells relative to controls

Initially, we analyzed the cell proliferation of A549 cells cultured aerobically in complete medium with 10% fetal bovine serum and 20 mM glucose. Previously, others reported that A549 cells resisted growth inhibition by 100 μM Prima-1 under normoxic conditions [31]. Now, we observed a limited response of A549 cells to 50 μM Prima-1 or 150 μM 3-BrPA after 48 h treatments in physiological 5 mM glucose [7]. However, both agents cooperated to suppress A549 cell proliferation. In contrast, 150 μM of the monocarboxylate transporter inhibitor, alpha-cyano-4-hydroxy-cinnamate (CHC) [34] did not increase Prima-1 toxicity (Fig. 1a). End-point semi-quantitative PCR and western blot were carried out with cells treated for shorter intervals than those used for inhibition of cell proliferation, since early morphological changes were seen following Prima-1 treatment (not shown). These experiments revealed a marked inhibition of SLC2A1-GLUT1 mRNA and diminished GLUT1 protein expression normalized to GAPDH in A549 cells treated with 50 μM Prima-1 (Fig. 1b). Essentially similar results were obtained in experiments in which cells were similarly treated but in the presence of 20 mM glucose (not shown).

### p21CDKN1A gene expression is increased by Prima-1 but not by 3-BrPA in A549 cells

Since Prima-1 is known to be a p53 reactivator [3, 6, 7], and the cyclin-dependent kinase inhibitor p21CDKN1 is a p53-activated gene promoting the G1 checkpoint control [35, 36] we confirmed by qPCR that Prima-1 increased expression of the p21CDKN1A mRNA in 5 mM or 20 mM glucose in A549 cells. However, this was antagonized by concomitant treatment with 3-BrPA (Fig. 1c), known to induce cell cycle arrest in S phase and G2/M [37]. The reciprocal effects of Prima-1 and 3-BrPA on p21CDKN1A expression may be related to the fact that they act at different cell cycle positions. It was of interest that the Prima-1 mediated increase in p21CDKN1A occurred in normoxia, hypoxia or in the presence of the hypoxia mimetic CoCl_2_ (Fig. 1d).

### Prima-1 cooperates with 3-BrPA to increase ROS

**Fig. 2:**
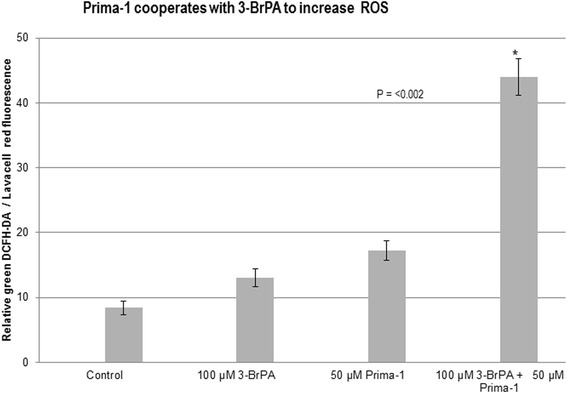
Prima-1 and 3-BrPA cooperate to increase ROS. ROS intracellular generation was assayed in octuplicates in adherent A549 cells seeded in 96 well plates 10 h after exposure to the indicated treatments in medium supplemented with 5 mM glucose, 2 mM glutamine and 5% dialyzed serum. This was quantitated using DCFH-DA (Life Technologies), a cell permeable non-fluorescent compound that can be hydrolyzed by intracellular esterases to DCFH, which fluoresces *green* when oxidized by H_2_O_2_. Cells were exposed to 20 μM DCFH-DA together with 20 μM LavaCell (Active Motif. Carlsbad, California 92008, USA) for 30 min. The latter is also a cell-permeable, non-toxic compound that stains membranes of live cells providing an *orange-red* emission (560–580 nM). Cell-associated fluorescence was determined using the signal thresholding algorithms identify fluorescence above the solution background from which fluorescent cells are identified for calculation of morphological and fluorescent parameters in an Isocyte argon laser spectrofluorometer identifying channel 1 green fluorescence (510–540) normalized to channel 3 orange-red cell fluorescence (560–580 nm). *denotes significance between treated cells relative to controls

Since 3-BrPA antagonized p21CDKN1A mRNA induction by Prima-1 (Fig. 1c), and 50 μM Prima-1 and 150 μM 3-BrPA cooperated to increase A549 cell inhibition (Fig. 1a) this suggested that induction of p21CDKN1A was not the major mechanism involved in the potentiation of toxicity by these agents. Based on reports that single treatment with Prima-1 [6, 8, 10–12] or with 3-BrPA [24, 25] increased ROS production, we investigated whether this effect was additive. For this, ROS production derived from the intracellular esterase processing of the cell-permeant 2',7'-dichlorodihydrofluorescein diacetate (DCFH-DA, Life Technologies, Carlsbad, Ca.) was quantitated cytofluorometrically, by measuring the normalized mean green fluorescence intensity. This showed that ROS production was essentially doubled after a 9 h treatment with both of these agents, prior to any evidence of overt toxicity which required 48 h treatments (Fig. 2). Since Prima-1 can alkylate p53 thiol groups [6] and 3-BrPA is another alkylating agent capable of increasing metabolic stress by generating free radicals [24, 25], results in Fig. 2 suggests that potentiation of oxidative stress is likely to mediate the synergy between 50 μM Prima-1 and 150 μM 3-BrPA, rather than only p53 activation.

### NAC counteracts toxicity of Prima-1 and 3-BrPA in (G12S)-mutant KRAS-A549 cells

**Fig. 3:**
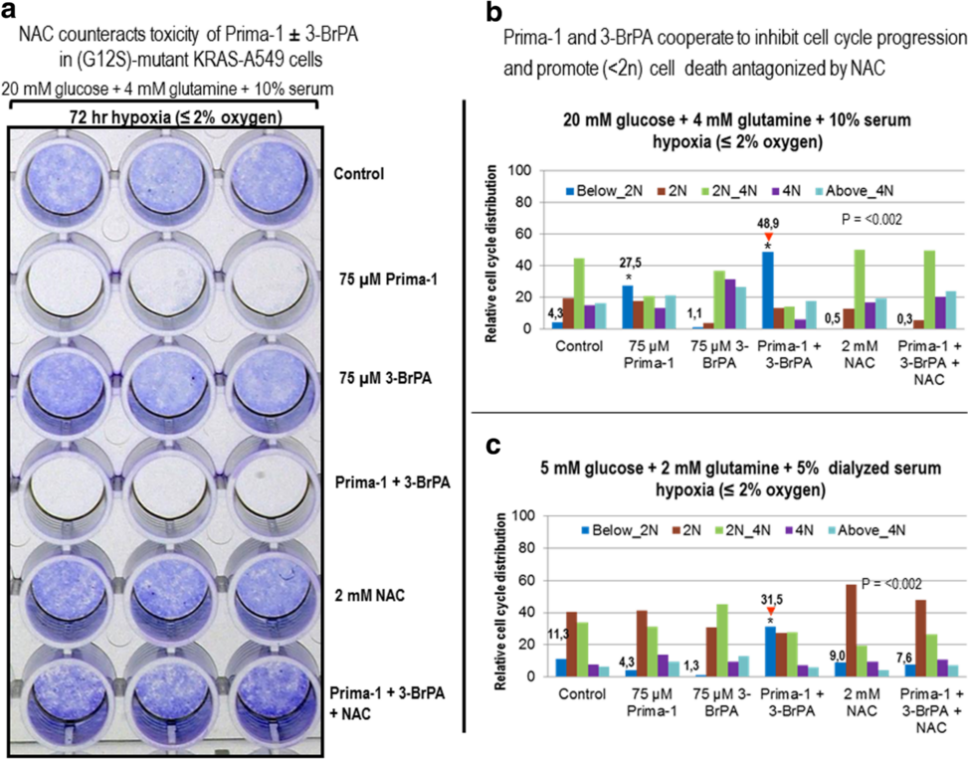
**a** NAC counteracts toxicity of Prima-1 and 3-BrPA in hypoxic (G12S)-mutant KRAS-A549 cells. Crystal violet staining of surviving cells was used to compare the response to a 72 h treatment with Prima-1 or 3-BrPA in A549 cultures under hypoxia in complete Dulbecco’s medium containing 20 mM glucose, 4 mM glutamine supplemented with 10% serum. **b** and **c** Prima-1 and 3-BrPA cooperate to inhibit cell cycle progression and promote hypoxic cell death is antagonized by NAC. Cell cycle analysis and assay of below_2n dead cells was performed as indicated under Methods for cells cultured under hypoxia for 48 h in 20 mM glucose, 4 mM glutamine supplemented with 10% serum, or 5 mM glucose, 2 mM glutamine and 5% dialyzed serum. *denotes significance between treated cells relative to controls

Based on the results shown in Fig. 2 we used the anti-oxidant N-acetylcysteine (NAC) to investigate whether NAC scavenging antagonized the effects of Prima-1 and 3-BrPA. Crystal violet survival studies revealed that Prima-1 was toxic as a single agent after 72 h of hypoxia against A549 cells in 20 mM glucose + 4 mM glutamine + 10% serum, which were not affected by 3-BrPA. However, Prima-1 toxicity against hypoxic A549 cells was counteracted by NAC even when added together with 3-BrPA (Fig. 3a).

### Prima-1 and 3-BrPA cooperation to inhibit cell cycle progression and promote hypoxic (<2n) cell death is antagonized by NAC

Cell cycle analysis was used to determine the influence of glucose supplementation and the consequences of 48 h treatments with Prima-1, 3-BrPA or NAC, under hypoxia. In 20 mM glucose and 4 mM glutamine, control cells and those treated with NAC were mostly in S phase (2n-4n). Cell cycle progression decreased reciprocally with an augmentation of the below_2n dead cell population [32] with single Prima-1 treatment and even more when this treatment was accompanied by 3-BrPA, effect reverted by NAC (Fig. 3b). In contrast, in 5 mM glucose, 2 mM glutamine and 5% dialyzed serum, there was an increase in the below_2n dead cell population when both Prima-1 and 3-BrPA were added to hypoxic A549 cells (Fig. 3c).

### GLUT1 is preferentially decreased by Prima-1 and ALDH1A1 is decreased by Prima-1 or 3-BrPA in 5 mM glucose in A549 cells

**Fig. 4:**
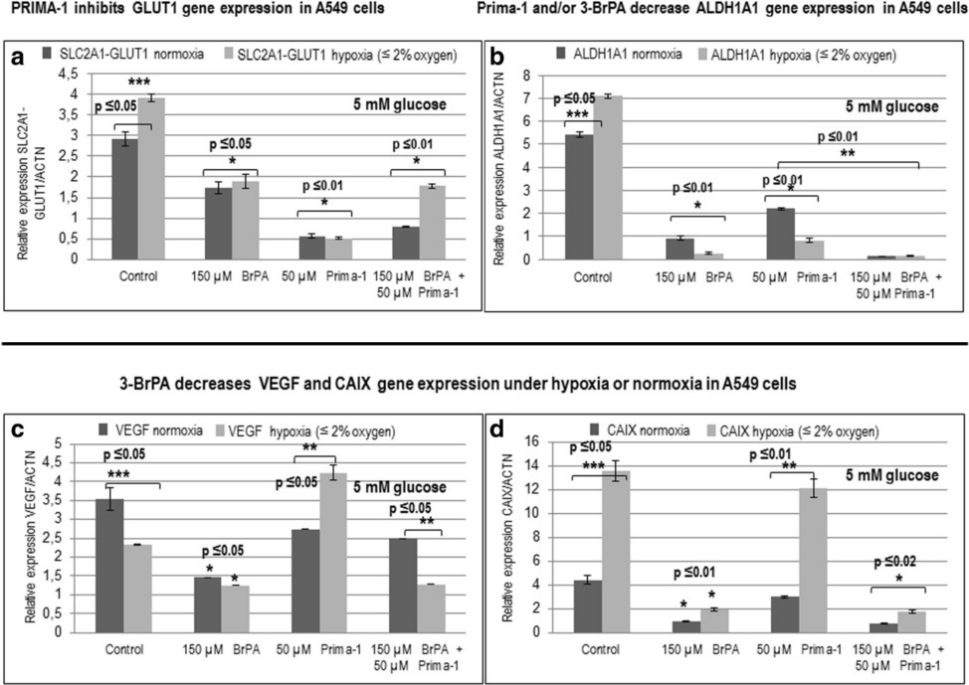
**a** Prima-1 lowers SLC2A1-GLUT1 gene expression in 5 mM glucose. A549 cells exposed to 5 mM glucose, 2 mM glutamine and 5% dialyzed serum, were kept for 4 h in hypoxia whenever indicated, followed by RNA isolation, and RT-qPCR, to assay SLC2A1-GLUT1 and ACTN gene expression bv qPCR, as indicated under Methods. **b** Decrease in ALDH1A1 induced by 3-BrPA is potentiated by Prima-1. Cells treated as indicated above were exposed to hypoxia whenever indicated, followed by RNA isolation, and RT-qPCR, to assay ALDH1A1 and ACTN gene expression bv RT- qPCR. **c** 3-BrPA counteracts Prima-1 hypoxic induction of VEGF gene expression. Cells treated as indicated above were exposed to hypoxia whenever indicated, followed by RNA isolation, and RT-qPCR, to assay VEGF and ACTN gene expression by RT- qPCR. **d** 3-BrPA suppresses CAIX gene expression. Cells seeded as indicated above were exposed to hypoxia for 4 h whenever indicated, followed by RNA isolation, and RT-qPCR, to assay CAIX and ACTN gene expression by q PCR. *denotes significance between treated cells relative to controls. **denotes significance between unequal treatments. ***denotes significance between hypoxia and normoxia

We also investigated the contribution of 3-BrPA to the regulation of GLUT1 and ALDH1A1 in physiological 5 mM glucose including Prima-1 when indicated. This revealed the preferential SLC2A1-GLUT1 decrease by Prima-1, partly attenuated by 3-BrPA particularly under hypoxia (Fig. 4a). In contrast, 3-BrPA preferentially down-regulated ALDH1A1 mRNA and this was not attenuated by Prima-1 (Fig. 4b).

### Prima-1 mediated enhancement of CAIX and VEGF under hypoxia in 5 mM glucose is antagonized by 3-BrPA in A549 cells

Expression of CAIX and VEGF mRNAs was not lowered significantly by Prima-1, as evidenced by qPCR. However, the expression of these 2 hypoxia-induced genes was diminished by 3-BrPA (Fig. 4c and d).

### Prima-1 and 3-BrPA cooperate to preferentially decrease LDHA rather than GAPDH in hypoxic A549 cells in 5 mM glucose

**Fig. 5:**
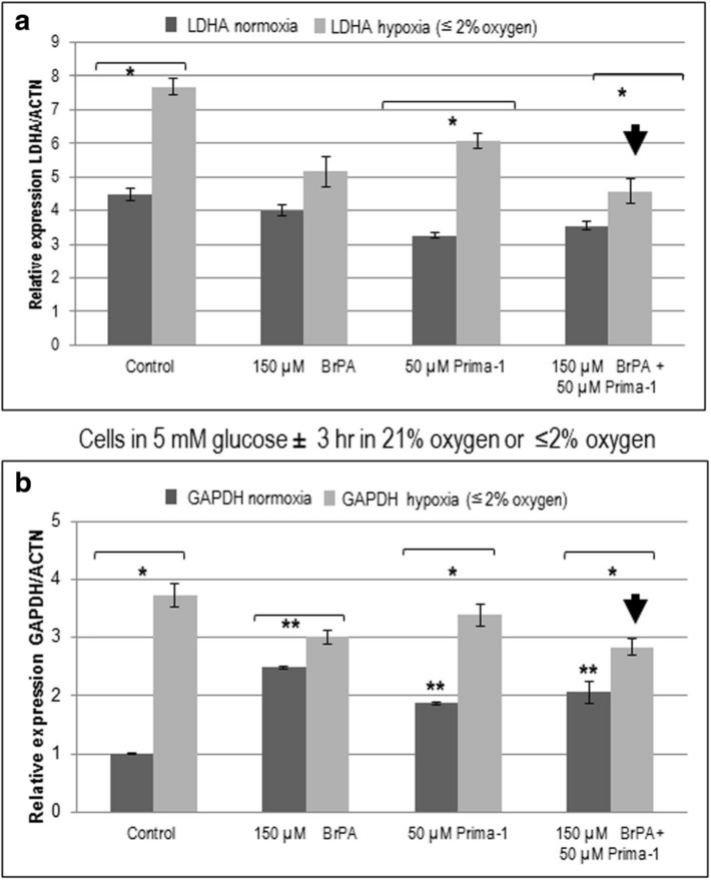
Prima-1 and 3-BrPA preferentially decrease hypoxia-induced LDHA rather than GAPDH in A549 cells. RT-qPCR was used to assay the effect of 3-BrPA and Prima-1 on LDHA and GAPDH gene expression under normoxia or 3 h under hypoxia. Note that combined use of Prima-1 and 3-BrPA lowers hypoxic LDHA expression in about 40%, in contrast to only an approximate 25% decline in hypoxic GAPDH expression by the same combined treatment. *denotes significance between hypoxic and normoxic cells. **denotes significance between treated and control cells

Since LDHA [38] and GAPDH [39] are also involved in glycolysis and hypoxia, their modulation by Prima-1 and/or 3-BrPA was investigated under normoxia or hypoxia in 5 mM glucose. Although mRNA expression of both genes was increased by hypoxia, Prima-1 and 3-BrPA preferentially cooperated to decrease hypoxia-induced LDHA rather than hypoxia-induced GAPDH (Fig. 5a and b).

### Prima-1 and 3-BrPA decrease GLUT1 external localization under hypoxia in A549 cells

**Fig. 6:**
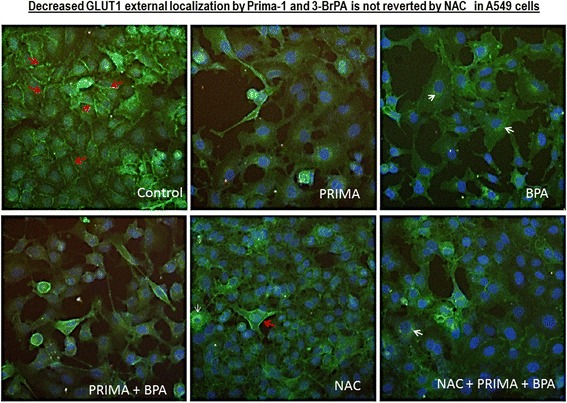
Decreased GLUT1 external localization by Prima-1 and 3-BrPA exposed for 3 h to hypoxia in A549 cells is not reverted by 2 mM NAC. Inmmune fluorescence was used to analyze preferential cell surface (*red arrows*) or perinuclear localization (*white arrows*) in p-formaldehyde fixed and permeabilized cells, treated as indicated in each case under hypoxia

Since cell-surface localization of the GLUT1 receptor is expected to promote glucose uptake [40], immune fluorescence localization of GLUT1 was compared in hypoxic control cells and in those treated with Prima-1, 3-BrPA, and/or the anti-oxidant N-acetylcysteine NAC, whenever indicated. This revealed preferential surface fluorescence essentially in control cells (Fig. 6).

### Hypoxic resistance to 3-BrPA in mutant KRAS C8161 cells is counteracted by Prima-1

**Fig. 7:**
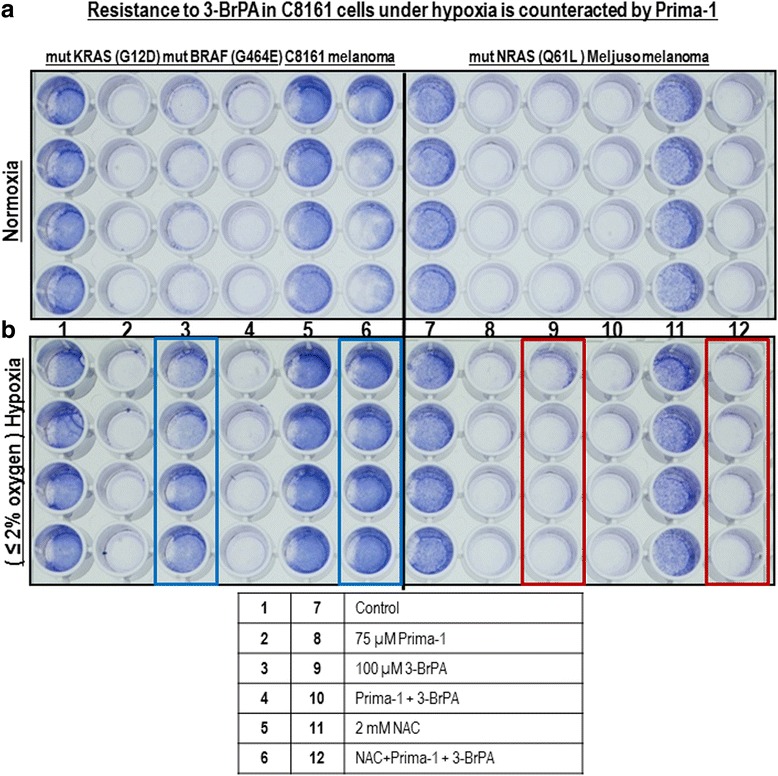
Resistance to 3-BrPA exposed for 4 h to hypoxia is counteracted by Prima-1 in C8161 cells. 5 x 10^3^ C8161 and MelJuso cells were allowed to adhere for 18 h in complete Dulbecco’s medium with 20 mM glucose, 4 mM glutamine and 10% fetal bovine serum, and subsequently exposed to the indicated treatments in the same medium for 72 h, followed by 3 h of hypoxia or normoxia. After washing twice in phosphate buffered saline pH7.4, adherent cells were fixed in 70% ethanol and surviving cells were evidenced by crystal violet staining **a**) normoxic cells; **b**) hypoxic cells

The susceptibility to Prima-1, 3-BrPA and NAC in MelJuso cells (BRAF wild-type (wt) and NRAS-Q61L mutant) [29] was compared with that of C8161 melanoma with an enhancing KRAS G12D mutation and a G464E mutation in the BRAF P loop region [31]. Under normoxia, both cell types were highly suceptible to either 75 μM Prima-1 or 100 μM 3-BrPA. However, NAC only protected C8161 cells from treatment with Prima-1 and 3-BrPA (Fig. 7a). Under hypoxia, susceptibility to Prima-1 persisted in both cell types but NRAS-mutant MelJuso cells showed greater susceptibility to 3-BrPA, compared to C8161 cells. Another difference between these 2 cell types was that 2 mM NAC counteracted the toxicity of Prima-1 and 3-BrPA only in hypoxic C8161 cells with no comparable attenuation against MelJuso cells (Fig. 7b). The NAC results counteracting the toxicity of Prima-1 and 3-BrPA also imply that this is mediated by oxidative stress, as shown for A549 cells (Figs. 2 and 3).

### Unequal modulation of glycolysis-hypoxia associated gene expression in MelJuso and C8161 cells

**Fig. 8:**
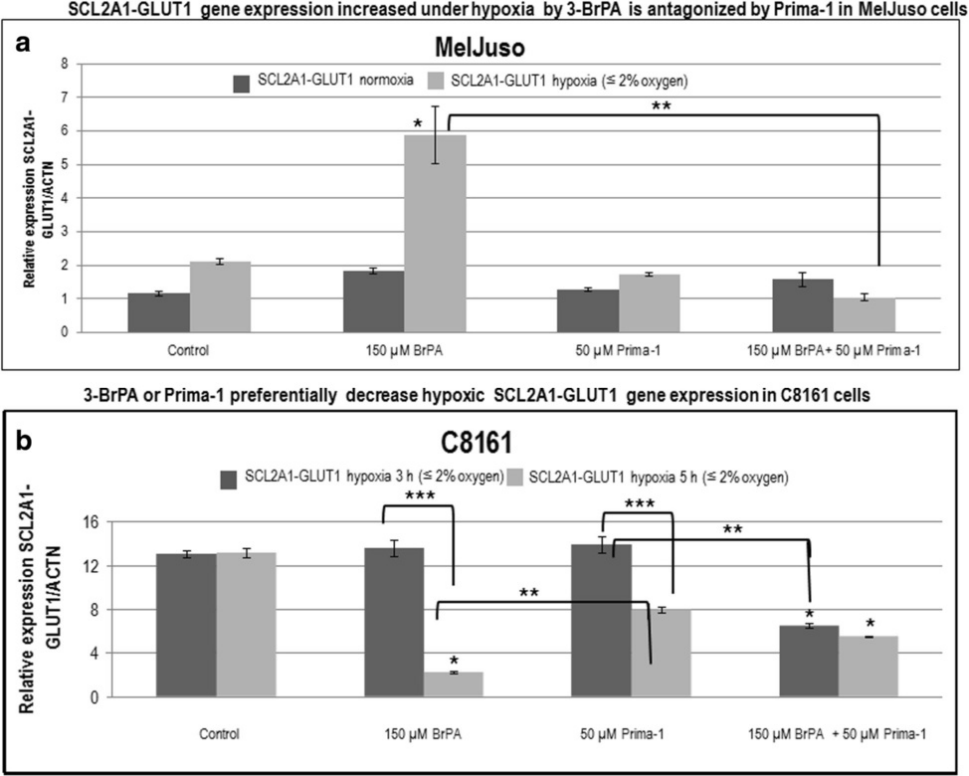
**a** SCL2A1-GLUT1 gene expression increased under hypoxia by 3-BrPA is antagonized by Prima-1 in MelJuso cells . RT-qPCR was used to assay the effect of 3-BrPA and Prima-1 on SLC2A1-GLUT1 normalized to ACTN gene expression under normoxia or 3 h of hypoxia in MelJuso cells, treated as indicated in Fig. 4a. Note that combined use of Prima-1 and 3-BrPA lowers SLC2A1-GLUT1 expression induced by 3-BrPA after 3 h hypoxia in MelJuso cells. **b** 3-BrPA or Prima-1 preferentially decrease hypoxic SCL2A1-GLUT1 gene expression in C8161 cells. RT-qPCR was used to assay the effect of 3-BrPA and Prima-1 on SLC2A1-GLUT1 normalized to ACTN gene expression under 3 or 5 h of hypoxia in C8161 cells, treated as indicated in Fig. 4a. Here, Prima-1, 3-BrPA or both treatments lowered SLC2A1-GLUT1 gene expression. *denotes significance between treated cells relative to controls. ** denotes significance between unequal treatments. ***denotes significance between 3 h and 5 h hypoxia

**Fig. 9:**
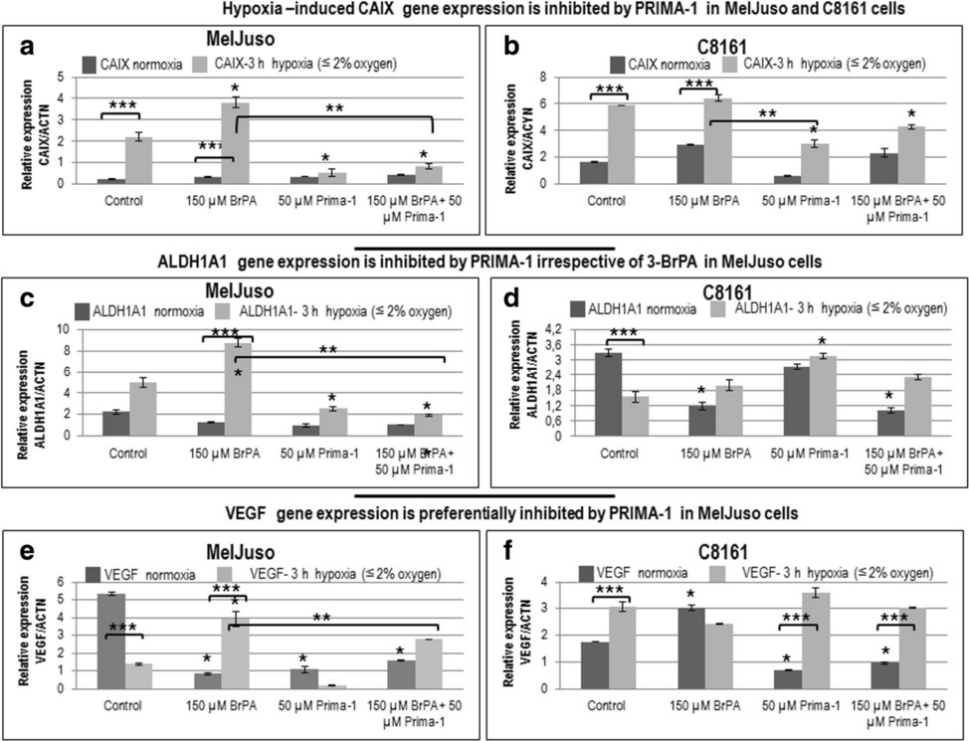
Modulation of CAIX, ALDH1A1 and VEGF mRNA expression by Prima-1 ± 3-BrPA. Cells cultured as indicated in Fig. 4a was used for RT-qPCR to assay the effect of 3-BrPA and Prima-1 on CAIX, ALDH1A1 and VEGF normalized to ACTN gene expression under normoxia or 3 h of hypoxia: **a** and **b** Hypoxia –induced CAIX gene expression is inhibited by Prima-1 in MelJuso and C8161 cells. **c** and **d** ALDH1A1 gene expression is preferentially inhibited by Prima-1 irrespective of 3-BrPA in MelJuso cells compared to C8161 cells. **e** and **f** VEGF gene expression is preferentially inhibited by Prima-1 in MelJuso cells compared to C8161 cells. *denotes significance between treated cells relative to controls. **denotes significance between unequal treatments. ***denotes significance between hypoxia and normoxia

Since 3-BrPA decreased VEGF and CAIX and Prima-1 inhibited SLC2A1-GLUT1 gene in KRAS-mutant A549 cells under hypoxia (Fig. 3), these parameters were also investigated by RT-qPCR in MelJuso and C8161 cells. When normalized to actin mRNA levels, control MelJuso cells showed much lower SLC2A1-GLUT1 expression than C8161 cells under comparable conditions. Although 3-BrPA increased the SLC2A1-GLUT1 expression in MelJuso cells, this was attenuated by Prima-1. Neither 3-BrPA nor Prima-1 lowered significantly SLC2A1-GLUT1 expression in C8161 cells under normoxia (not shown). However, SLC2A1-GLUT1 expression was diminished by 3-BrPA, Prima-1 or both treatments after 5 h of hypoxia in C8161 cells (Fig. 8a, b). Although CAIX gene expression was inhibited by Prima-1 in MelJuso and C8161 cells (Fig. 9a and b), ALDH1A1 and VEGF gene expression were inhibited by Prima-1 only in MelJuso cells (Fig. 9).

## Discussion

**Fig. 10:**
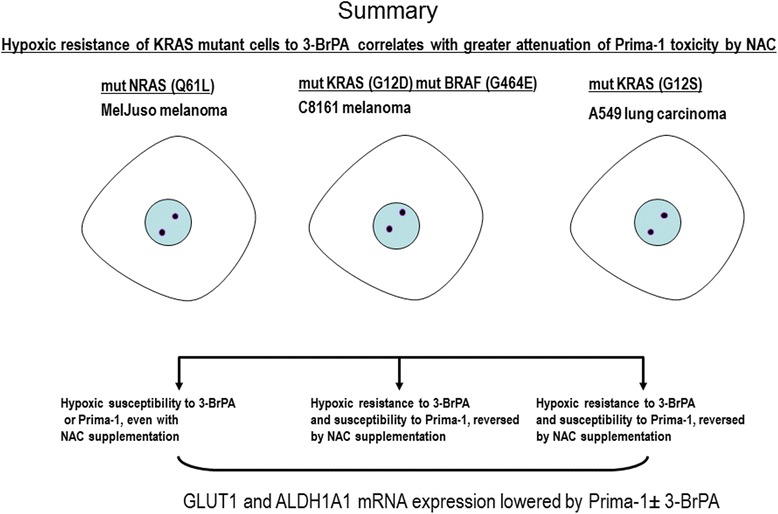
SUMMARY NRAS. NRAS-mutant cell hypoxic susceptibility to Prima-1 ± 3-BrPA is not reversed by 2 mM NAC. Hypoxic resistance of KRAS mutant cells to 3-BrPA correlates with greater attenuation of Prima-1 toxicity by 2 mM NAC

3-BrPA is a pro-oxidant and alkylating anti-tumor agent capable of inhibiting glycolytic and mitochondrial targets and generating free radicals [17, 24, 25]. 3-BrPA at 110 μM was shown to suppress the growth of colorectal carcinoma cells with KRAS or BRAF mutations surviving glucose starvation [26]. In contrast, we showed in aerobic ERα positive breast cancer cells, that wt p53 conferred resistance to 3-BrPA, since p53 silencing, or use of genetically matched cells with mutant p53 R175H, revealed high susceptibility to 75 μM 3-BrPA [41]. The wt p53-induced resistance to 3-BrPA was independently confirmed in RT4 (grade I; wild-type *p53*) bladder cancer cells that remained unaffected by 125 μM 3-BrPA, in contrast to T24 (grade III; mutant *p53*) bladder cancer cells, which greatly diminished their survival at comparable 3-BrPA concentrations [42]. This report further investigated how to enhance 3-BrPA toxicity against wt p53 A549 non-small cell lung cancer cells harbouring a KRAS G12S gene mutation. These A549 cells also showed poor susceptibility to 3-BrPA (Figs. 1 and 2). Others previously reported poor susceptibility to 100 μM Prima-1 in aerobic A549 cells in RPMI 1640 medium containing 11 mM glucose plus any sugar contained in non-dialyzed 10% fetal bovine serum [31]. Since basal oxidative stress is increased by mutant p53 [13] or mutant KRAS [14], and can be further exacerbated by 3-BrPA [25] or Prima-1 [12], we showed for the first time that the latter 2 agents cooperate to hyper-induce ROS under aerobic conditions (Fig. 2) and counteract hypoxic resistance to 3-BrPA using physiological 5 mM glucose levels. Although KRAS mutant cells over-express glucose transporter-1 (SLC2A1-GLUT1) [14] with some of the glucose directed to attenuate excessive oxidative stress through the generation of NADPH by the pentose phosphate pathway [14–16], we showed that Prima-1 decreased GLUT-1 expression in A549 cells (Fig. 1b) and in C8161 cells (Fig. 5c), result compatible with that of other p53-reactivating molecules [43]. Moreover, 50 μM Prima-1 also synergized with 150 μM 3-BrPA rather than with 150 μM CHC to inhibit A549 aerobic cell proliferation (Fig. 1a). Under hypoxia, A549 cells were also resistant to 150 μM 3-BrPA but susceptible to 75 μM Prima-1, when used as single agents (Fig. 3a). The toxicity caused by Prima-1 + 3-BrPA under hypoxia involved excessive ROS, since it was reversed by the glutathione precursor, NAC (Fig. 3a). The 72 h crystal violet assay after hypoxic treatment with 75 μM Prima-1 (Fig. 3a) is compatible with reports of Prima-1 anti-tumor activity under hypoxia irrespective of p53 status [8, 11]. A cytofluorometric cell cycle analysis [32] after a shorter 48 h hypoxic treatment confirmed that Prima-1 was more effective than 3-BrPA as a single agent against KRAS-mutant A549 but also showed that 3-BrPA cooperated to inhibit cell cycle progression and promote (<2n) cell death antagonized by NAC in these cells (Fig. 3b and c). NAC prevented Prima-1 ± 3-BrPA toxicity against hypoxic KRAS G12S-mutant A549 cells (Fig. 3) and KRAS G12D-mutant C8161 cells (Fig. 7). In contrast, hypoxic NRAS-(Q61L)-mutant MelJuso melanoma cells were greatly susceptible to 3-BrPA or Prima-1, but were not protected by NAC (Fig. 7b). Unequal response to NAC may be linked to lower endogenous anti-oxidant glutathione in hypoxic NRAS-(Q61L)-mutant MelJuso susceptible cells, and lower basal SCL2A1-GLUT1 mRNA expression normalized to actin mRNA, compared to that seen in the 3-BrPA resistance in hypoxic C8161 cells (Fig. 8). In our studies, cells were seeded in complete Dulbecco’s medium containing ***20 mM*** glucose supplemented with undialyzed 10% serum for 24 h. followed by washing 3X with PBS and treated as indicated in each case in medium supplemented with physiological 5 mM glucose and 5% dialyzed serum. The transition from 20 to 5 mM glucose together with a hypoxic (≤ 2% oxygen) possibly mimicked a restrictive glucose condition [44]. The latter affects inducibility of HIF*-*1α and some of the genes induced by hypoxia, which requires not only low oxygen but significant glucose availability [44–47]. This may explain the lower VEGF expression in 5 mM glucose in hypoxic A549 (Fig. 4c) and MelJuso cells (Fig. 9e), resembling results showing lower VEGF-A transcripts in mouse pancreas beta cells after hypoglycemia [48] No comparable decrease was seen in VEGF or CAIX mRNA expression in hypoxic C8161 cells (Fig. 9f), implying that the response to hypoxia in 5 mM glucose may be tumor cell-dependent. A similar glucose concentration also enhanced 3-BrPa-induced cell death in colorectal carcinoma Lovo and HT-29 cells, which was suppressed at 20 mM glucose, concomitantly with down-regulation of the hMCT1 bromopyruvate carrier [44]. Our findings that hypoxia increases resistance to 3-BrPA in KRAS-mutant wt p53 tumor cells rather than in those with NRAS mutation, and the reversal of this resistance with Prima-1 is important, because no effective single clinical therapy has been consistently achieved to treat tumors linked to KRAS [28, 37, 38] or NRAS mutations [28, 40, 49–51]. The greater susceptibility to Prima-1 ± 3-BrPA in hypoxic mutant NRAS MelJuso melanoma (Summary, Fig. 10), suggests that agents like Prima-1 + 3-BrPA, may help attenuate the frequent acquisition of resistance to targeted therapy against V600E BRAF-mutated tumors which acquire NRAS mutations [52].

### Conclusions

This report compared tumor cells with KRAS or NRAS mutations, because those with HRAS constitute only 1–3% of human cancers [53], with KRAS or NRAS mutations being more frequent. Mutated KRAS induces GLUT1 [26, 39] and stem cell-like properties in human cancer [54] linked to ALDH1A1 expression [18, 19]. Prima-1 by itself or in combination with 3-BrPA down-regulates these genes and also lowers CAIX [20, 21] and VEGF mRNA expression [55]. Addressing the question of toxicity of this combination treatment on normal cells, Prima-1 analog PRIMA-1^Met^ shows limited cytotoxicity toward normal hematopoietic cells while decreasing multiple myeloma cell viability irrespective of p53 status [56]. Similarly, 3-BrPA does not affect non-malignant cells [57] being incorporated into glycolytic tumor cells through the monocarboxylate transporter hMCT1 [58] known to be down-regulated by high glucose [44]. Hence, resistance of hypoxic KRAS-mutant tumor cells to 3-BrPA through lower hMCT1 is likely to be antagonized by lower glucose uptake linked to diminished GLUT-1 transporter expression, a p53 function [59] now shown to be reactivated by Prima-1. Taken together, this is the first report showing that Prima-1 overcomes the resistance to 3-BrPA in hypoxic wt p53 KRAS-mutant cells [8, 28, 33, 60] by promoting wt p53 reactivation [61] and pro-oxidant cancer therapeutics [9, 12, 62].

## Highlights

- Hypoxia increases resistance to 3-bromopyruvate (3-BrPA) in KRAS-mutant wt p53 cells
- Prima-1, a p53 reactivator decreases GLUT1 and counteracts hypoxic resistance to 3-BrPA
- N-acetylcysteine reverts toxicity induced by Prima-1 and 3-BrPA

## Abbreviations

3-BrPA: 3-bromopyruvate
CAIX: Carbonic anhydrase 9
ROS: Reactive oxygen species
SLC2A1-GLUT1: Glucose transporter 1
VEGF: Vascular endothelial growth factor.
